# Safety of TNF-blocking agents in rheumatic patients with serology suggesting past hepatitis B state: results from a cohort of 21 patients

**DOI:** 10.1186/ar2868

**Published:** 2009-11-26

**Authors:** Caroline Charpin, Sandrine Guis, Philippe Colson, Patrick Borentain, Jean-Pierre Mattéi, Patrice Alcaraz, Nathalie Balandraud, Benoit Thomachot, Jean Roudier, René Gérolami

**Affiliations:** 1 Service de Rhumatologie, Centre Hospitalier Universitaire Conception, 147 Boulevard Baille, 13385 Marseille, France; 2 Laboratoire de Virologie, Fédération Hospitalière de Microbiologie Clinique, Centre Hospitalo-Universitaire Timone, 264 Rue Saint-Pierre, 13385 Marseille, France; 3 URMITE CNRS-IRD UMR 6236, Facultés de Médecine et de Pharmacie, Université de la Méditerranée, 27 Boulevard Jean Moulin, 13385 Marseille, France; 4 Service d'Hépato-gastroentérologie, Centre Hospitalier Universitaire Conception, 147 Boulevard Baille, 13385 Marseille, France

## Abstract

**Introduction:**

Reactivation of hepatitis B virus (HBV) infection in patients with past infection has been described in 5% to 10% of individuals undergoing immunosuppressive therapies. No data are available to date on the outcome of patients treated by tumour necrosis factor-alpha (TNFα) inhibitors for chronic arthritis with a serological pattern of past HBV infection. The aim of our study was to monitor HBV markers in HBV surface antigen (HBsAg)-negative/anti-HBcAb-positive patients treated with a TNFα inhibitor for inflammatory arthritides.

**Methods:**

Twenty-one HBsAg-negative/anti-HBcAb-positive patients were included. HBV serological patterns were compared with those determined before starting TNFα inhibitors. Serum HBV DNA testing by polymerase chain reaction was additionally performed. Spearman correlation analysis was used and *P *< 0.05 was chosen as the significance threshold.

**Results:**

Before starting therapy, mean anti-HBsAb titre was 725 IU/L, no patient had an anti-HBsAb titre <10 IU/L, and 18 patients had an anti-HBsAb >100 IU/L. At a mean time of 27.2 months following therapy introduction, mean anti-HBsAb titre was 675 IU/L and anti-HBsAb titre remained >100 IU/L in 17 patients. There was a strong correlation between the first and second anti-HBsAb titres (*r *= 0.98, *P *= 0.013). Moreover, no patient had an anti-HBsAb titre below 10 IU/L or HBV reactivation (HBsAg seroreversion or positive HBV DNA detection). However, the anti-HBsAb titre decreased by more than 30% in 6 patients. The mean anti-HBsAb titre at baseline was significantly lower (*P *= 0.006) and the mean duration of anti-TNFα therapy, although non-significant (*P *= 0.09), was longer in these six patients as compared to patients without a decrease in anti-HBsAb titre.

**Conclusions:**

Anti-TNFα treatments are likely to be safe in patients with past hepatitis B serological pattern. However, the significant decrease of anti-HBsAb titre observed in a proportion of patients deserves HBV virological follow-up in these patients, especially in those with a low anti-HBsAb titre at baseline.

## Introduction

Hepatitis B virus (HBV) reactivation is a life-threatening disease that is known to occur in HBV inactive carriers following polychemotherapy or immunosuppressive treatments. Thus, HBV reactivation has been reported to occur in up to 50% of HBV surface antigen (HBsAg)-positive patients following polychemotherapy for haematological cancer [1], and in these patients, preventive anti-HBV therapy is recommended [2]. In addition, several studies have pointed out that HBV reactivation was possible, though at a much lower frequency, in patients undergoing immunosuppressive chemotherapy and whose HBV serological patterns indicate past hepatitis B, as defined by HBsAg negativity and anti-HBV core antibody positivity resulting in severe acute hepatitis and significant morbidity and mortality rates despite antiviral therapy.

Tumour necrosis factor-alpha (TNFα) inhibitors that are widely used in chronic inflammatory arthritides, inflammatory bowel diseases, and psoriasis treatment are likely to interfere with the natural history of chronic HBV infection. Production of TNFα has been shown to be elevated in the liver of patients chronically infected with HBV; TNFα participates in the clearance of HBV by promoting elimination of HBV-infected hepatocytes and inhibiting HBV replication. More recently, TNFα has been shown to play a key role in the control of the immune response directed against HBV. Thus, TNFα may inhibit the suppressive effect of regulatory T cells on the HBV-specific immune response and lack of TNFα induces impaired proliferation of HBV-specific cytotoxic T lymphocytes [3]. TNFα inhibitors are therefore likely to promote HBV replication and reactivation. In this view, some case reports have had a fatal outcome because of HBV reactivation following infliximab administration in HBsAg-positive patients [4-9]. In these patients, TNFα inhibitors should not be used without preventive anti-HBV therapy. Except for one case report [10], no data are available to date in the outcome of patients treated with TNFα inhibitors for chronic inflammatory arthritides with a serological pattern of past HBV infection, although this serological status is much more frequently encountered as compared with HBsAg positivity. In the present work, we aimed at detecting HBV reactivation in a cohort of patients with past HBV infection who underwent TNFα inhibitor treatment for chronic inflammatory rheumatism.

## Materials and methods

### Patients

#### Selection of anti-TNFα-treated patients and hepatitis B virus serological patterns

Five hundred four patients followed in the department of rheumatology were tested for hepatitis B serological pattern between 2005 and 2006. Of them, 284 had a totally negative serology, 2 (0.4%) had a serology indicating chronic hepatitis B (HBsAg positivity), and 58 (13%) had an HBV serology indicating spontaneously cured hepatitis B (HBsAg-negative, anti-HBcAb-positive, ± anti-HBeAb-positive); 54 of these 58 patients were anti-HBsAb-positive and the remaining 4 were anti-HBsAb-negative. In addition, 8 patients harboured isolated anti-HBcAb (without anti-HBsAb or anti-HBeAb). Finally, 152 patients had a serological pattern in agreement with HBV vaccination (isolated anti-HBsAb positivity). Twenty-four of the 58 patients with a serology indicating cured hepatitis B were treated for rheumatoid arthritis (RA) or spondylarthropathy by one or more anti-TNFα. Three of them could not be included in this study: one died, one withdrew consent, and one was lost to follow-up. Finally, a total of 21 patients gave informed consent for the study and were included.

### Methods

Peripheral blood analyses, including blood count, transaminase activity (aspartate transaminase [AST] and alanine transaminase [ALT]), gamma-glutamyl transferase [GGT], bilirubin dosage, and hepatitis B serological pattern before anti-TNFα treatment and during follow-up, were compared. The mean duration between the two blood samples was 35 months (range 12 to 60 months). Additionally, HBV DNA detection using polymerase chain reaction (PCR) was performed in the last serological pattern determination.

#### Detection of hepatitis B viral DNA

Quantification of viral DNA was performed using a real-time PCR assay (Cobas Ampliprep/Cobas TaqMan 48 assay; Roche Diagnostics, Meylan, France). The detection threshold was 12 IU/L (1.08 log IU/mL, or 70 copies/mL).

#### Anti-HBs antibody dosage

Anti-HBsAb quantification was performed using the Axsym Abbott immunoenzymatic assay (Abbott Diagnostics, Wiesbaden, Germany). An anti-HBsAb titre of less than 10 IU/L was considered negative. An anti-HBsAb titre of greater than 100 IU/L was considered to represent good protective immune status in a healthy subject, even if it is admitted that a greater than 10 IU/L titre is sufficient for conferring a protective effect against HBV after immunization [11]. HBsAg and anti-HBcAb were detected using Axsym Abbott immunoenzymatic assays.

### Statistical analysis

The difference of anti-HBsAb titre between the first and second serum samples was analysed using a non-parametric test: the Spearman rank correlation coefficient. A *P *value of less than 0.05 was chosen as the significance threshold. AST, ALT, and GGT levels and bilirubin dosage were considered to increase when there was twofold elevation.

## Results

### Baseline characteristics of the 21 patients included

The baseline characteristics of the 21 patients before the first blood test analysis are as follows: the majority of patients (62%) were women, patients had a mean age ± standard deviation of 57.7 ± 2.7 years (range 38 to 81), and the disease duration was 11 ± 2.2 years (range 1 to 30). Twelve patients suffered from RA, 5 from psoriasic arthritis, and 4 from ankylosing spondylarthritis (AS). At the end of the study, 4 patients were treated with infliximab, 14 with etanercept, and 3 with adalimumab. Thirteen patients received only one TNFα inhibitor: 8 were treated with etanercept, 3 with adalimumab, and 2 with infliximab. Because of non-response to one TNFα inhibitor, 6 patients had two types of TNFα inhibitors, and 2 patients did not respond to two TNFα inhibitors but benefited from three TNFα inhibitors. TNFα inhibitor dosage was usual: 50 mg per week for etanercept, 40 mg twice a month for adalimumab, and 3 to 5 mg/kg for 8 weeks (RA patients) or 6 weeks (AS patients) for infliximab. Ten of the 21 patients also received methotrexate (7.5 to 15 mg per week). The mean duration of treatment was 27.2 months (range 7 to 56).

### Hepatitis B serology, hepatitis B viral detection, and liver enzymes

#### Initial anti-HBsAb titre analysis

Three patients (one with RA and two with AS) had an anti-HBsAb titre of less than 100 IU/L at the first blood analysis: 16, 22, and 94 IU/L.

#### Anti-HBsAb titre evolution

**Figure 1:**
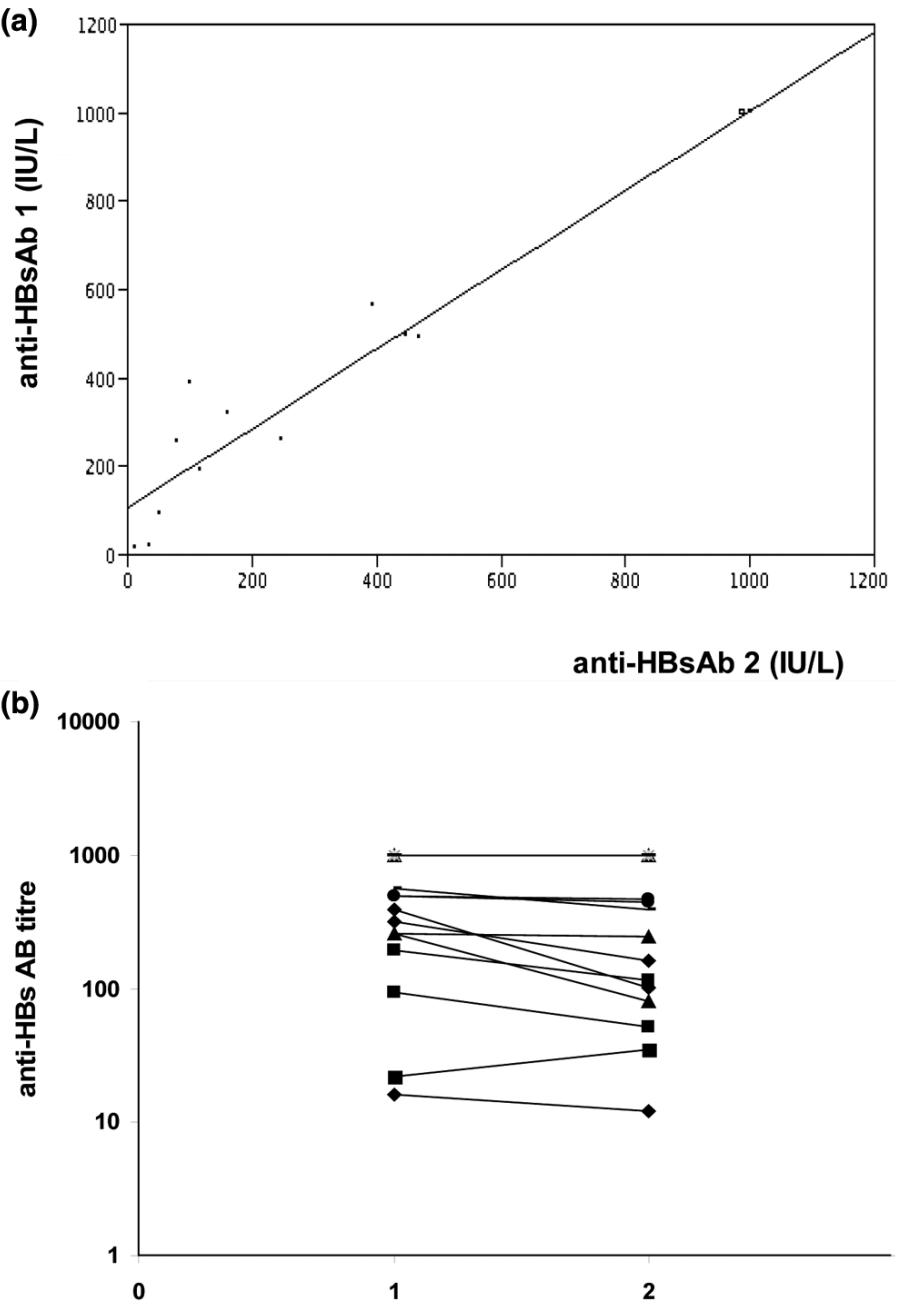
Correlation between group 1 and 2 anti-HBs Ab titre. (a) Correlation between anti-HBsAb titre of the first (baseline) and second (end of follow-up) dosages (anti-HBsAb 1 and anti-HBsAb 2, respectively). The coefficient of correlation is *r *= 0.98, and *P *= 0.013 by the Spearman rank correlation. (b) Evolution of anti-HBsAb titre for each patient between the first (1) and second (2) dosages.

The anti-HBsAb titre remained greater than 10 IU/L in all patients at the second blood analysis (the lowest titre was 12 IU/L). A tendency for the decrease of anti-HBsAb titre by an average of 48 IU/L (8%) in comparison with the first measurement was observed. No patient had an increase in anti-HBsAb titre. Before the start of therapy, the mean anti-HBsAb titre was 725 IU/L, and at the second sample, the mean anti-HBsAb titre was 675 IU/L. There was a strong correlation between the anti-HBsAb titre of the first and second blood samples (*r *= 0.98, *P *= 0.013) (Figure 1a, b). Only 1 of 21 patients experienced a decrease of anti-HBsAb level below 100 IU/L, from 256 to 80 IU/L. A subgroup of six patients experienced a significant decrease in anti-HBsAb titres, ranging from 30% to 70%. In these six patients, the mean anti-HBsAb titre at baseline was significantly lower (*P *= 0.006), and although it did not reach significance, the mean duration of anti-TNF therapy was longer (*P *= 0.09) as compared with patients who did not present a decrease in anti-HBsAb. Four of them had a therapeutic association with methotrexate (Table 1).

**Table 1:** Comparison of group 1 and group 2 patients

Patient characteristics	Group 1 (n = 6)	Group 2 (n = 15)
Mean age, years	56	57
Percentage of female patients	77	77
Percentage of patients with rheumatoid arthritis	77	53
Mean anti-HBsAb titre, IU/L	302	753
Mean duration of disease, years	13	10
Mean duration of treatment, months	34	24
Association with methotrexate (mean dosage: 10 mg/week)	4	6
Association with corticosteroid	1	4
Two successive anti-TNFα inhibitors	2	4
Three successive anti-TNFα inhibitors	0	2

Group 1 patients underwent a strong decrease in anti-HBsAb and group 2 patients did not. TNFα, tumor necrosis factor-alpha.

#### Hepatitis B virus reactivation

No hepatitis B reactivation, as defined by HBsAg or HBV DNA detection, was observed.

#### Liver enzyme evolution

At the beginning of the study, three patients had significant abnormalities of liver enzymes, even if only one of them had a methotrexate association. At the time of the second blood sample, only one patient (not one of the three previous patients) had significant abnormalities; she was treated with methotrexate. All patients had a normal platelet count.

## Discussion

The main result of the present work is that we did not observe HBV reactivation following TNFα inhibitor treatment in patients with a serological pattern of past HBV infection. This is a very important result. Indeed, although TNFα inhibitors are likely to interfere with the natural history of chronic HBV infection and HBV reactivation has been described in HBsAg-positive patients following TNFα inhibitors (including case reports with a severe or fatal outcome), no clear recommendation for the management of patients presenting a serological status of past HBV infection exists to date.

HBV reactivation in patients with serological tests of past HBV infection (that is, HBsAg-negative, anti-HBcAb-positive with or without anti-HBsAb) has been previously described following immunosuppressive therapies or polychemotherapy, especially those including biotherapies such as rituximab [12]. In these cases, HBV reactivation is explained by the persistence of HBV DNA inside the hepatocyte nucleus [13]. This allows the reappearance of HBV replication in a context of immune suppression [14]. The risk of HBV reactivation following TNFα inhibitors in such patients is therefore a concern and was recently underlined [4].

Moreover, this serological profile is frequently encountered. The rates of prevalence of this serological profile in the general population in France in 2004 were estimated to be 9.7% for men and 6.7% for women [15]. Thus, in the present study, 13% of the 504 patients from the department of rheumatology presented with an HBV serology indicating spontaneously cured hepatitis B.

The present work is the first to assess the safety of TNFα inhibitors in a cohort of patients with a serological pattern of past HBV infection, and although a larger number of patients should be necessary to draw a firm conclusion, it strongly suggests that the risk of TNFα inhibitor-related HBV reactivation is very low in these patients, even after several years of therapy. A longer follow-up of these patients, however, should be mandatory. Hence, although anti-HBsAb titre always remained higher than 10 IU/L and a very strong correlation between the first and second anti-HBsAb titres could be observed for each patient (*r *= 0.98, *P *= 0.013), a large decrease (30% to 70%) of anti-HBsAb titres could be noted in a significant proportion (6 of 21 patients) of TNFα inhibitor-treated patients, especially those presenting with a low anti-HBsAb at baseline. This point should be taken into consideration because such a decrease in anti-HBsAb titre has been shown to precede HBV reactivation in patients treated for haematological cancer or following immunosuppressive therapy [16], stressing the need for a closer follow-up of these patients. Although the difference did not reach significance, it is noteworthy that the mean duration of anti-TNFα therapy was longer in these six patients than in those in whom anti-HBsAb titre remained stable. A more prolonged follow-up of these patients will therefore be necessary. This decrease in anti-HBsAb titre might also be related to the underlying chronic disease itself as well as other coincidental factors such as concurrent immunosuppressive therapy. In this view, it is worth noting that four of six patients with a large decrease in anti-HBsAb titres were also treated with methotrexate. Further studies will also be necessary to assess the risk of HBV reactivation in patients undergoing biotherapies other than anti-TNFα treatment.

## Conclusions

Although further studies that include more patients with a longer follow-up are necessary, it is likely that anti-TNFα treatments can be safely used in patients treated for chronic inflammatory arthritides with an HBV serological pattern indicating past hepatitis B. Thus, no HBV reactivation was observed after a mean of 27 months of anti-TNFα treatment. In addition, anti-HBsAb titre remained above 10 IU/L for all patients. However, the decrease of anti-HBsAb titre observed in a significant proportion of patients suggests that HBV virological follow-up should be considered during anti-TNFα therapy in patients with past HBV infection.

## Abbreviations

Ab: antibody; ALT: alanine transaminase; AS: ankylosing spondylarthritis; AST: aspartate transaminase; GGT: gamma-glutamyl transferase; HBsAg: hepatitis B virus surface antigen; HBV: hepatitis B virus; PCR: polymerase chain reaction; RA: rheumatoid arthritis; TNFα: tumour necrosis factor-alpha.

## Competing interests

The authors declare that they have no competing interests.

## Authors' contributions

CC and SG participated in patient recruitment, data acquisition and data analysis and helped to draft the manuscript. PC participated in serological data acquisition and data analysis and helped to draft the manuscript. PB, J-PM, PA, NB, BT, and JR participated in data analysis and patient recruitment. RG conceived of the study and participated in its design and coordination. All authors read and approved the final manuscript.

## References

[B1] Blanpain C Knoop C Delforge ML Antoine M Peny MO Liesnard C Vereerstraeten P Cogan E Adler M Abramowicz D Reactivation of hepatitis B after transplantation in patients with pre-existing anti-hepatitis B surface antigen antibodies: report on three cases and review of the literature Transplantation 1998 66 883 886 10.1097/00007890-199810150-00012 9798698

[B2] Lalazar G Rund D Shouval D Screening, prevention and treatment of viral hepatitis B reactivation in patients with haematological malignancies Br J Haematol 2007 136 699 712 10.1111/j.1365-2141.2006.06465.x 17338776

[B3] Kasahara S Ando K Saito K Sekikawa K Ito H Ishikawa T Iseki H Shinozaki Y Kimura K Kuwabara E Koide S Nakazawa H Mori H Lack of tumor necrosis factor alpha induces impaired proliferation of hepatitis B virus-specific cytotoxic T lymphocytes J Virol 2003 77 2469 2476 10.1128/JVI.77.4.2469-2476.2003 12551985 PMC141095

[B4] Esteve M Saro C Gonzalez-Huix F Suarez F Forne M Viver JM Chronic hepatitis B reactivation following infliximab therapy in Crohn's disease patients: need for primary prophylaxis Gut 2004 53 1363 1365 10.1136/gut.2004.040675 15306601 PMC1774200

[B5] Millonig G Kern M Ludwiczek O Nachbaur K Vogel W Subfulminant hepatitis B after infliximab in Crohn's disease: need for HBV-screening? World J Gastroenterol 2006 12 974 976 16521231 10.3748/wjg.v12.i6.974 PMC4066168

[B6] Ostuni P Botsios C Punzi L Sfriso P Todesco S Hepatitis B reactivation in a chronic hepatitis B surface antigen carrier with rheumatoid arthritis treated with infliximab and low dose methotrexate Ann Rheum Dis 2003 62 686 687 10.1136/ard.62.7.686 12810441 PMC1754595

[B7] Wendling D Auge B Bettinger D Lohse A Le Huede G Bresson-Hadni S Toussirot E Miguet JP Herbain G Di Martino V Reactivation of a latent precore mutant hepatitis B virus related chronic hepatitis during infliximab treatment for severe spondyloarthropathy Ann Rheum Dis 2005 64 788 789 10.1136/ard.2004.031187 15834064 PMC1755487

[B8] Kaur PP Chan VC Berney SN Histological evaluation of liver in two rheumatoid arthritis patients with chronic hepatitis B and C treated with TNF-alpha blockade: case reports Clin Rheumatol 2008 27 1069 1071 10.1007/s10067-008-0896-y 18521652

[B9] Sakellariou GT Chatzigiannis I Long-term anti-TNFalpha therapy for ankylosing spondylitis in two patients with chronic HBV infection Clin Rheumatol 2007 26 950 952 10.1007/s10067-006-0392-1 16865308

[B10] Raftery G Chronic viral hepatitis and TNF blockade Rheumatology (Oxford) 2007 46 1381 10.1093/rheumatology/kem082 17567635

[B11] West D Vaccine induced immunologic memory for hepatitis B surface antigen: implications for policy on booster vaccination Vaccine 1996 14 1019 1027 10.1016/0264-410X(96)00062-X 8879096

[B12] Yeo W Chan TC Leung NW Lam WY Mo FK Chu MT Hui ED Lei KI Mok TS Chan PK Hepatitis B virus reactivation in lymphoma patients with prior resolved hepatitis B undergoing anticancer therapy with or without rituximab J Clin Oncol 2009 27 605 611 10.1200/JCO.2008.18.0182 19075267

[B13] Zoulim F New insight on hepatitis B virus persistence from the study of intrahepatic viral cccDNA J Hepatol 2005 42 302 308 10.1016/j.jhep.2004.12.015 15710212

[B14] Listing J Strangfeld A Kery S Rau R von Hinueber U Stoyanova-Scholz M Gromnica-Ihle E Antoni C Herzer P Keko WJ Schneider M Zink A Infections in patients with rheumatoid arthritis treated with biologic agents Arthritis Rheum 2005 52 3403 3412 10.1002/art.21386 16255017

[B15] Meffre C Prevalence of hepatitis B in France, 2003-2004 J Hepatol 2006 44 S22 10.1016/S0168-8278(06)80047-4

[B16] Wands JR Chuna CM Roll FJ Maddrey WC Serial studies of hepatitis-associated antigen and antibody in patients receiving antitumor chemotherapy for myeloproliferative and lymphoproliferative disorders Gastroenterology 1975 68 105 112 1054319

